# Vasodilatory effects of glucagon: A possible new approach to enhanced subcutaneous insulin absorption in artificial pancreas devices

**DOI:** 10.3389/fbioe.2022.986858

**Published:** 2022-09-21

**Authors:** Ingrid Anna Teigen, Misbah Riaz, Marte Kierulf Åm, Sverre Christian Christiansen, Sven Magnus Carlsen

**Affiliations:** ^1^ Department of Clinical and Molecular Medicine, Faculty of Medicine and Health Sciences, Norwegian University of Science and Technology, Trondheim, Norway; ^2^ Department of Endocrinology, St. Olav’s Hospital, Trondheim University Hospital, Trondheim, Norway

**Keywords:** glucagon, pharmacokinctics, subcutaneous infusion, artificial pancreas, diabetes mellitus type 1

## Abstract

Patients with diabetes mellitus type 1 depend on exogenous insulin to keep their blood glucose concentrations within the desired range. Subcutaneous bihormonal artificial pancreas devices that can measure glucose concentrations continuously and autonomously calculate and deliver insulin and glucagon infusions is a promising new treatment option for these patients. The slow absorption rate of insulin from subcutaneous tissue is perhaps the most important factor preventing the development of a fully automated artificial pancreas using subcutaneous insulin delivery. Subcutaneous insulin absorption is influenced by several factors, among which local subcutaneous blood flow is one of the most prominent. We have discovered that micro-doses of glucagon may cause a substantial increase in local subcutaneous blood flow. This paper discusses how the local vasodilative effects of micro-doses of glucagon might be utilised to improve the performance of subcutaneous bihormonal artificial pancreas devices. We map out the early stages of our hypothesis as a disruptive novel approach, where we propose to use glucagon as a vasodilator to accelerate the absorption of meal boluses of insulin, besides using it conventionally to treat hypoglycaemia.

## Graphical Abstract

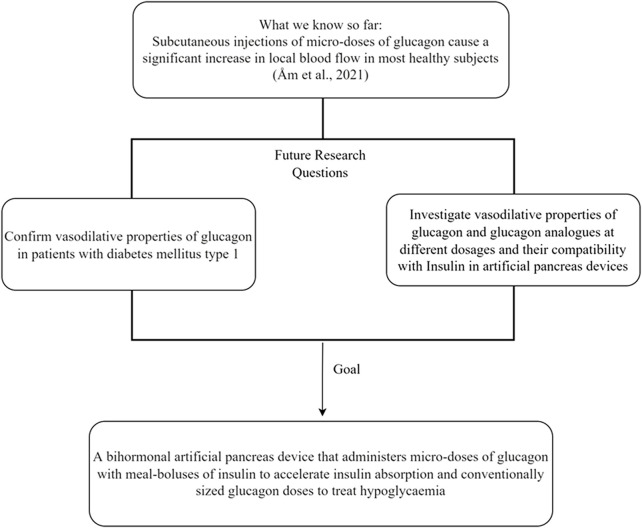

## 1 Introduction

Patients with diabetes mellitus type 1 (DM1) have lost their ability to produce and secrete insulin due to the selective destruction of beta cells in the pancreatic islets of Langerhans. Insulin is necessary for a wide range of physiological processes and is mandatory for keeping blood glucose concentrations within a narrow range. Thus, patients with DM1 depend entirely on exogenous insulin, and intensive insulin treatment and tight glucose control are recommended to reduce the risk of microvascular complications (Reichard, 1992; Nathan et al., 1993; Reichard et al., 1996). Most patients with DM1 use multiple daily subcutaneous (SC) insulin injections or continuous SC insulin infusions administered via a pump. Recently, double SC artificial pancreas devices have also been introduced as a treatment option, i.e., devices that measure SC glucose concentrations continuously and calculate and deliver suitable insulin doses automatically (Peyser et al., 2014). Currently, only hybrid artificial pancreas devices that require meal annunciations from the user are commercially available (Knebel and Neumiller, 2019). As they require frequent user intervention, patients using these hybrid devices are not fully relieved of the daily focus and stress of managing their disease, which is known to be a factor that causes distress and reduced mental well-being among patients with DM1 (Pallayova and Taheri, 2014).

Administering appropriate meal-time insulin doses is a major daily challenge for patients with DM1, and delayed or miscalculated insulin dosages often results in postprandial glucose excursions and poor glucose control (Robinson et al., 2021). Timing and adjusting insulin dosages is difficult because all insulins, even the most rapid-acting insulin analogues, are absorbed relatively slowly from the SC tissue, with an onset of effect after 10–30 min and a maximum glucose-lowering effect one to 3 hours after administration (AHFS Drug Information, Insulins General Statement, 2022a). Whereas in healthy individuals, postprandial blood glucose concentrations have returned to baseline within the same period (Hiyoshi et al., 2017).

Patients with DM1 are advised to inject meal-time insulin 10–20 min before they start eating to accommodate the insulin absorption delay. However, despite this precaution, glucose concentrations are often strongly elevated after meals (Birkeland, 2006). If fully automated artificial pancreas devices that require no user intervention are to become a reality, this issue must be resolved, as such devices evidently must detect ingestion of a meal before increasing insulin infusion.

## 2 Factors delaying onset of insulin action in SC artificial pancreas devices

A fully automated SC artificial pancreas device may only upscale the insulin infusion rate in relation to a meal once a meal is detected by a rise in the SC glucose concentration or through another sensing modality. As such, two major barriers exist that prevent the development of such a device: The delay in sensing changes in blood glucose concentrations from a sensor in SC tissue and, more importantly, the delayed absorption, and hence the glucose-lowering effect of SC delivered insulin (Christiansen et al., 2017; Gingras et al., 2018).

### 2.1 Glucose sensing in SC tissue

SC continuous glucose monitoring systems (CGM) report glucose concentrations in the interstitial fluid in SC tissue (Funtanilla et al., 2019). Changes in blood glucose concentration are not immediately followed by a corresponding change in CGM measurements, as glucose must first diffuse through the capillary walls and into the interstitial space. This process leads to sensing delay, particularly during rapid changes in blood glucose concentrations, such as during physical activity and after meals (Schmelzeisen-Redeker et al., 2015; Funtanilla et al., 2019).

Although there have been considerable improvements in CGM-technology in recent years, the sensing delay is still an issue that needs to be resolved. In the literature, the delay is commonly attributed to both physiological and technological factors and is estimated to be in the range of 5–10 min (Schmelzeisen-Redeker et al., 2015; Siegmund et al., 2017).

### 2.2 Insulin absorption from SC tissue

When administered SC compared to intravenously, the half-life of human insulin increases from approximately 6 min to 3 h (Binder, 1969; Skjaervold et al., 2012), predominantly because of the significant delay in absorption from SC tissue (Lindholm and Jacobsen, 2001). SC absorption of insulin is influenced by several factors, contributing to considerable interindividual and intraindividual variation. Of particular importance are physical-chemical factors related to the drug type, physiological factors on the administration site, such as skin temperature and SC blood flow, and differences in absorption kinetics in the anatomical regions suitable for SC injections (Gradel et al., 2018; Pitt et al., 2020).

#### 2.2.1 Drug type

Soluble human insulin comprises oligomers, primarily monomers, dimers, and hexamers, in chemical equilibrium. When insulin is stored at high concentrations and in the presence of allosteric ligands, the oligomer equilibrium shifts towards a very large fraction of hexamers. This process ensures long shelf-life of insulin formulations, as hexamers are more resistant to chemical degradation (Gast et al., 2017). However, insulin hexamers are too large to be absorbed by the SC capillaries and must dissociate before getting absorbed into the systemic circulation and to become biologically active as insulin monomers (Gradel et al., 2018).

Rapid-acting insulin analogues, which are used in artificial pancreas devices, follow a similar pattern. Both insulin aspart, insulin glulisine, and insulin lispro are structurally identical to human insulin except for minor alterations in the amino-acid sequences that exist to reduce the tendency of insulin monomers to form larger oligomers in SC tissue (AHFS Drug Information, Insulin aspart, Insulin glulisine, Insulin lispro, 2022b). Thus, the rapid-acting insulin analogues are more easily absorbed, resulting in a faster onset and shorter duration of action (Gradel et al., 2018).

#### 2.2.2 Insulin concentration and volume

Highly concentrated insulin formulations are usually absorbed more slowly from SC tissue than diluted preparations. However, smaller injection volumes cause less pain to the patient (Zijlstra et al., 2018) and additionally have a larger surface-to-volume ratio, which promotes absorption (Søeborg et al., 2009; Mader et al., 2013; Gradel et al., 2018).

#### 2.2.3 Anatomical site and injection technique

There is a considerable variation in insulin absorption rate from different anatomical areas. Absorption is reported to be fastest from the SC tissue on the abdomen, followed by the arms, and lowest from the thighs and gluteal region (Bantle et al., 1993). Absorption may also vary within the areas, and rotation between and within injection regions is a likely source for intraindividual variability in insulin pharmacokinetics (Gradel et al., 2018).

#### 2.2.4 SC blood flow

Variations in skin blood circulation are essential for thermoregulatory control and can differ substantially depending on the presence of vasoactive substances such as noradrenaline and nitric oxide (Charkoudian, 2003).

Insulin monomers and dimers are absorbed directly into SC capillaries through simple diffusion. When the capillary exchange surface in SC tissue expands with the recruitment of capillaries, vasodilation and increased blood flow, insulin absorption is accelerated. As such, insulin absorption is positively correlated to the blood flow at the injection site (Binder, 1969; Vora et al., 1992) and can be promoted through injection into areas with hyperperfusion, for instance, after direct or indirect heating or skin massage (El-Laboudi and Oliver, 2015).

The ultra-rapid insulin lispro formulation, Lyumjev^®^ (Eli Lilly) and the fast-acting insulin aspart formulation Fiasp^®^ (Novo Nordisk) contain vasoactive additives that increase blood flow and accelerate the absorption of the insulin analogue. Lyumjev^®^ contains the vasodilating drug treprostinil, a stable prostacyclin analogue, and citrate in addition to insulin lispro (The Norwegian Medicines Agency Medicine Database, Lyumjev, 2022). This combination leads to significantly faster SC absorption, elimination, and earlier onset of action of insulin lispro (Leohr et al., 2021). Fiasp^®^ contains niacinamide and l-arginine together with insulin aspart. Niacinamide increases the relative fraction of insulin monomers in SC tissue and causes transient local vasodilation after administration, leading to increased initial absorption of insulin aspart (Kildegaard et al., 2019).

#### 2.2.5 Individual factors

Obesity, age, gender, smoking, and comorbidities associated with DM1 all contribute to extensive interindividual variation. In addition, absorption may also be delayed or decreased by the presence of insulin-binding antibodies, which develop in nearly all patients a few months after the start of insulin treatment (AHFS Drug Information: American Society of Health-System Pharmacists, 2022e).

## 3 The fear of hypoglycaemia and the need for a bihormonal approach

Although intensive insulin treatment is recommended to reduce long-term microvascular complications in patients with DM1, it increases the risk of severe hypoglycaemia, an acute and dramatic condition that can be life-threatening if left untreated (Nathan et al., 1993). The fear of hypoglycaemia significantly reduces the quality of life for patients with DM1 and prevents tight glucose control (Chatwin et al., 2021).

In healthy individuals, the alpha cells in the pancreatic islets of Langerhans secrete glucagon as a response to declining blood glucose concentrations to stimulate hepatic glucose output and prevent hypoglycaemia. In patients with DM1, this pancreatic response is reduced or even absent, which makes them susceptible to hypoglycaemic episodes (Gerich et al., 1973).

SC bihormonal artificial pancreas devices that utilise both insulin and glucagon can potentially allow more aggressive insulin treatment than unihormonal devices using only insulin, as glucagon could counteract insulin-induced hypoglycaemia.

Studies comparing bihormonal and unihormonal SC artificial pancreas devices have reported that the bihormonal approach reduces the incidence and duration of daytime hypoglycaemic episodes. However, evidence of long-term improvements in the time spent in the normoglycaemic range and effects on HbA1c is lacking (Bakhtiani et al., 2013; Weisman et al., 2017; Haidar, 2019).

## 4 Vasodilatory effects of SC micro-doses of glucagon

For decades, it has been known that large doses of glucagon have physiologic effects beyond increasing glucose output. For instance, it may reduce gastrointestinal motility, increase heart contractility and cause vasodilation (Farah, 1983).

Glucagon has previously been shown to cause up to a 500 per cent increase in SC blood flow after SC injection of a large dose (1 mg) (Simmons and Williams, 1992). Our research group recently discovered that SC injections of much smaller doses of glucagon (0.1 and 0.01 mg) could also cause a substantial increase in local SC blood flow (Åm et al., 2022). In this study, glucagon was injected SC on the abdomen in healthy individuals, and blood flow was evaluated by laser doppler technology. The median increase in SC blood flow was 250 per cent after using a dosage of 0.1 mg glucagon. The blood flow peaked at 2–4 min after injection before declining slowly, and the effect was still present 30 min after injection. Although injection of glucagon caused increased SC blood flow in most of the study subjects, the effect was not observed in seven of the 22 included participants, which were classified as non-responders (Åm et al., 2022).

The effect of glucagon on SC blood flow appears to be local and is not present 2 cm lateral to the glucagon injection site as evaluated by both laser doppler technology and a thermal camera (unpublished data). This observation is perhaps unsurprising, as SC blood supply is organised vertical to the skin’s surface, with arterioles situated 1.5–7 mm apart depending on the area of the body (Braverman et al., 1992; Raju et al., 2012).

Unlike insulin, glucagon is absorbed rapidly after SC administration, reaching maximum plasma concentrations in humans at approximately 10–20 min (Simmons and Williams, 1992; Graf et al., 1999; El-Khatib et al., 2010; El Youssef et al., 2014; Blauw et al., 2016; Castle et al., 2016; Ranjan et al., 2016; Hövelmann et al., 2018; Hövelmann et al., 2019). Previous animal trials conducted by our research group demonstrated no significant difference in the absorption speed or glucose elevating effect of glucagon after SC compared to intraperitoneal administration (Dirnena-Fusini et al., 2018; Åm et al., 2020; Teigen et al., 2022). This observation contrasts with the major delay in insulin absorption and effect after SC administration compared to intraperitoneal administration (Christiansen et al., 2017). Therefore, we hypothesise that the local vasodilatory effect of glucagon promotes its absorption from the SC space, as this would explain why there is no significant difference in absorption or the time to onset and maximum effect of SC versus intraperitoneally delivered glucagon (Teigen et al., 2022).

## 5 Discussion: A new approach for bihormonal artificial pancreas devices

As previously addressed, it is probably impossible to achieve excellent glucose control by a fully automated SC artificial pancreas device without any feature that can enhance insulin absorption.

If it is confirmed in future trials that micro-doses of glucagon increase local SC blood flow in patients with DM1, this effect can be utilised in SC bihormonal artificial pancreas devices where tubes that deliver insulin and glucagon could lie in immediate proximity (i.e., the hormones are delivered via a dual-lumen line). Micro-doses of glucagon could be given at the same time as meal boluses of insulin to promote postprandial insulin absorption and larger doses of glucagon may be administered when indicated to treat and prevent hypoglycaemia.

The glucagon dosages that have been used previously in SC bihormonal artificial pancreas devices to counteract hypoglycaemia resemble those we have used to demonstrate increased SC blood flow in healthy subjects (Haidar, 2019). An obvious objection to using glucagon to enhance insulin absorption is that glucagon exerts antagonistic pharmacodynamic effects on glucose metabolism to insulin and may increase blood glucose concentrations, which is highly undesirable in the postprandial state. However, in studies investigating the potential benefit of adding glucagon to artificial pancreas devices, glucagon has been administered to prevent hypoglycaemia and not with insulin infusions or in relation to meals. Glucagon’s ability to increase blood glucose concentrations seems to be influenced by the concentration of circulating insulin, and it is most effective in situations with impending hypoglycaemia and low systemic insulin concentrations (Castle et al., 2010; Bakhtiani et al., 2015). This is the opposite of the situation in which we suggest using micro-doses of glucagon to increase blood flow, where insulin concentrations are increasing.

Further, there is evidence supporting a meal-induced glucagon release from the intestine in subjects with DM1 (Cryer, 2012; Knop, 2018; Yosten, 2018; Bengtsen and Moller, 2021; Ito et al., 2021). This glucagon release appears within minutes of oral glucose or food ingestion. Glucagon originating from the intestines is drained directly via the portal system to the liver, where it might increase glucose output. Results from a previous study by our research group support a saturable first-pass metabolism of glucagon in the liver (Teigen et al., 2021). Therefore, we hypothesise that micro-doses of glucagon administered with meal-time insulin would probably have no additional effects on postprandial glucose excursions as the liver will already be saturated with glucagon from the intestine.

In the study by Åm et al., there was a substantial proportion of non-responders to glucagon (Åm et al., 2022). The extent of interindividual variation and the mechanisms causing non-response are not yet known and should be elucidated in future studies. Individual response to glucagon will need to be confirmed before using it as part of a patient’s artificial pancreas algorithm.

## 6 Future perspectives and research

We hypothesise that bihormonal SC artificial pancreas devices may use micro-doses of glucagon to induce local vasodilation and accelerate SC insulin absorption after meals. We suggest investigating the effects of micro-dosages of glucagon that are administered together with insulin boluses via a dual-lumen delivery line by the artificial pancreas when it detects a meal. The glucagon dose should be the minimum dose required to achieve adequate vasodilation while the insulin dose should be adjusted according to the effect on glucose levels. The same glucagon delivery line may also be used to deliver conventionally sized doses of glucagon during hypoglycaemic events when the insulin infusion rate is low or discontinued.

Human glucagon is relatively unstable and may aggregate and form amyloid fibrils quite rapidly, depending on the solution’s temperature and pH-value. Therefore, human glucagon is recommended to be used within 24 h after reconstitution in artificial pancreas devices, although there is some evidence indicating that reconstituted human glucagon could be chemically stable for up to 7 days (El-Khatib et al., 2007; Taleb et al., 2017). The stable glucagon analogue dasiglucagon was approved for clinical use by the FDA in 2021, but it has not yet been granted marketing authorisation in Europe (AHFS Drug Information, Dasiglucagon Hydrochloride, 2022d). Questions regarding the vasodilative properties of different glucagon formulations, dosages of glucagon or glucagon analogues to be used, chemical compatibility with different insulin preparations and technical details of the glucagon delivery in an artificial pancreas device are obvious subjects for future animal trials and clinical research in patients with DM1.

## 7 Conclusion

The recent discovery of a pronounced local vasodilative effect of SC administered micro-doses of glucagon should be studied further in patients with DM1. If confirmed, a disruptive change in the use of glucagon in patients with DM1 may soon emerge as micro-doses of glucagon could be used to accelerate the absorption of meal boluses of insulin from SC tissue. This could potentially improve the performance of SC bihormonal artificial pancreas devices. Thus, a fully automated bihormonal artificial pancreas that can provide excellent glucose control without daily input from the patient may be achievable.

## Data Availability

The original contributions presented in the study are included in the article/supplementary material, further inquiries can be directed to the corresponding author.
